# Elevated 1-****α**** Hydroxylase Activity in Monocytes from Patients with Active Tuberculosis

**DOI:** 10.1155/2013/928138

**Published:** 2013-11-25

**Authors:** Yi-Ching Tung, Tsan-Teng Ou, Wen-Chan Tsai

**Affiliations:** ^1^Department of Public Health and Environmental Medicine, College of Medicine, Kaohsiung Medical University, 100 Tzyou 1st Road, Kaohsiung 807, Taiwan; ^2^Graduate Institute of Medicine, College of Medicine, Kaohsiung Medical University, 100 Tzyou 1st Road, Kaohsiung 807, Taiwan; ^3^School of Medicine, College of Medicine, Kaohsiung Medical University, 100 Tzyou 1st Road, Kaohsiung 807, Taiwan; ^4^Department of Internal Medicine, Kaohsiung Medical University Hospital, 100 Tzyou 1st Road, Kaohsiung 807, Taiwan; ^5^Laboratory Medicine, Kaohsiung Medical University Hospital, 100 Tzyou 1st Road, Kaohsiung 807, Taiwan

## Abstract

A uremic patient developed hypercalcemia after tuberculosis infection, and his ionized calcium levels correlated with 1,25-dihydroxyvitamin D_3_ (1,25(OH)_2_D_3_) levels. We performed further studies to determine whether monocytes are alternative sites of 1,25(OH)_2_D_3_ conversion beyond renal tubular cells. Using an ex vivo bioassay, in this study, we found that 1-**α** hydroxylase (CYP27B1) activity in monocytes is significantly higher in patients with active tuberculosis (TB) than in those with frequent TB contact. However, when monocytes from patients with active TB were restimulated with antigen derived from *Mycobacterium tuberculosis*, less 1,25(OH)_2_D_3_ was observed. In contrast, the level of 1,25(OH)_2_D_3_ was unchanged in those with frequent TB contact. We conclude that monocytes may be an alternative source of 1-**α** hydroxylase that could convert 25-hydroxyvitamin D_3_ to the more active 1,25(OH)_2_D_3_.

## 1. Introduction

Tuberculosis has plagued the world since prehistoric times. According to a recent report, one million patients succumb to tuberculosis and related comorbidities annually [1]. In recent decades, tremendous effort has been made to understand the pathophysiological process of the disease. Animal models and human studies have paved the way for the clarification of individual immune responses to *Mycobacterium tuberculosis* (MTB) [2–5]. These studies provide evidence that vitamin D plays an important role in human resistance to tuberculosis. vitamin D has been used to fight tuberculosis for more than 200 years. Cod liver oil and calciferol, major sources of vitamin D, have been used since the 17th century to treat patients with tuberculosis [6]. In normal physiology, after sunshine exposure, 7-dehydrocholesterol stored in the skin was converted to previtamin D_3_ followed by thermal isomerization to vitamin D_3_ [7]. Next, vitamin D_3_ is hydroxylated in the liver to 25-hydroxyvitamin D_3_ (25(OH)D_3_). Then, 25(OH)D_3_ is further hydroxylated to the most active form of vitamin D_3_, 1,25(OH)_2_D_3_, by 1-*α* hydroxylase (CYP27B1) [8, 9]. Renal tubular epithelial cells are a major source of 1-*α* hydroxylase and play a critical role in determining the concentration of 1,25(OH)_2_D_3_ in the serum [10]. The presence of CYP27B1 in extrarenal tissues has been recognized for decades [11]. The impact of extrarenal CYP27B1 on the serum concentration of 1,25(OH)_2_D_3_ has yet to be determined.

**Figure 1:**
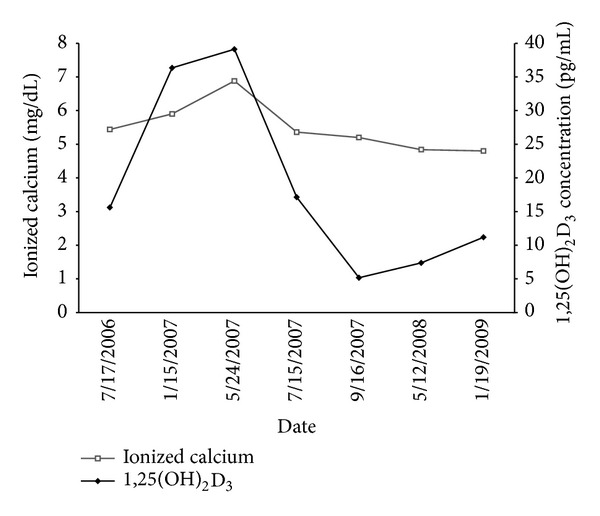
Changes in ionized calcium and 1,25(OH)_2_D_3_ concentrations in the serum of a patient with active TB and uremia.

We encountered a 34-year-old patient with uremia who had received regular hemodialysis for 2 years. The patient experienced muscle weakness with fatigue for 2 days before visiting a family physician. Blood chemistry revealed elevated ionized calcium levels (5.44 mg/dL). Despite the hypercalcemia, the patient's symptoms were relieved by conventional treatment. Four months later, the patient experienced progressive muscle weakness accompanied by fever. In the ER, elevated ionized calcium levels were again noted (6.88 mg/dL). In addition, an EKG revealed an abnormal heart rhythm and a short QT interval with widened T wave. A chest X-ray showed right upper lobe pulmonary infiltration that was later determined to be pulmonary tuberculosis. The patient then received 9 months of antituberculosis treatment. Four months posttreatment, the patient's ionized calcium levels normalized (5.2 mg/dL), with a complete resolution of symptoms. To elucidate the pathogenesis of this patient's hypercalcemia, we measured 1,25(OH)_2_D_3_ levels in addition to ionized calcium levels. As shown in Figure 1, the levels of 1,25(OH)_2_D_3_ were highly correlated with those of ionized calcium.

In addition to renal CYP27B1 expression, macrophages and monocytes are considered important extrarenal sites of CYP27B1 expression. In this study, we evaluated the role of monocytes in the metabolism of vitamin D_3_ using an ex vivo bioassay. Furthermore, we stimulated monocytes with antigen derived from MTB to obtain further insight into how monocytes modulate vitamin D_3_ metabolism in response to bacterial challenge.

## 2. Materials and Methods

### 2.1. Subject Population

This study was performed at Kaohsiung Medical University. Participants were stratified into two groups: (1) active TB and (2) frequent TB contact. Those with active pulmonary tuberculosis confirmed by a sputum culture and chest film were assigned to the active TB group (*n* = 25). Frequent TB contacts (*n* = 25) included the following: (1) medical staff who had worked at the TB center for at least 10 years and had never been infected and (2) TB patients' family members, clinicians, and nurses who had long-term contact with TB patients and had never been infected. All frequent TB contacts had been vaccinated with BCG and underwent a yearly chest X-ray; when chest films were abnormal, a Mantoux test was performed. We excluded subjects with diabetes, malignancy, or any other disease that could cause immunodeficiency. The two groups were sex and age matched (Table 1).

### 2.2. Cell Preparation

Peripheral blood mononuclear cells (PBMCs) were isolated from heparin-treated blood collected from the 2 groups of donors using a standard Ficoll-Paque (Pharmacia, Uppsala, Sweden) gradient. PBMCs (1 × 10^6^ cells/mL) were resuspended in RPMI 1640 medium supplemented with L-glutamine and 10% fetal calf serum. After incubation for 1 hour in a 75 cm^2^ flask in a humidified 37°C, 5% CO_2_ incubator, the medium containing nonadherent cells was decanted into a conical tube, and the flask was washed twice with serum-free medium to remove any residual nonadherent cells. Adherent monocytes were removed by gentle scraping with a plastic cell scraper. The cells were transferred to a conical tube, centrifuged to remove the wash solution, and resuspended to 3 × 10^6^ cells/mL in supplemented medium. The adherent cell population contained more than 85% CD14^+^ cells. 

### 2.3. Antigen

MTB isolated from patients with tuberculosis was resuspended in phosphate-buffered saline and heat-killed in a water bath at 70°C for 70 minutes. The bacteria were then sonicated using a New Highway sonicator (Farmingdale, NY, USA). The protein concentration of the bacterial homogenate was determined using the Pierce BCA protein assay kit (IL, USA) and stored at −20°C. 

### 2.4. Cytofluorometric Analysis

 Cytometric analysis was performed using a FACS cytometer (Becton Dickinson). A total of 3 × 10^5^ cells were incubated with each monoclonal antibody in saturating quantities in 50 *μ*L of staining buffer (Hank's balanced salt solution: 1% BSA and 0.1% sodium azide) for 1 hour at room temperature and then washed three times with PBS. The cells were prepared for analysis by suspension in 500 *μ*L of 1% paraformaldehyde in PBS. The monocyte population was gated for analysis based on a side-scatter and forward-scatter dot plot. A total of 5,000 gated cells were used for each analysis. Gated cells were further analyzed by fluorescence staining using fluorescein-isothiocyanate- (FITC-) conjugated anti-CD14 (Ancell). 

### 2.5. Quantitation of 1,25(OH)_2_D_3_

 Purified monocytes were cultured at a density of 3 × 10^5^ cells/mL in 100 *μ*L in each well of a 96-well tissue culture plate with 200 nM 25(OH)D_3_ dissolved in a final concentration of 1% ethanol. After 3 hours of incubation, 1 mL of acetonitrile was added to stop the reaction. The cells and medium were harvested and combined with an equal volume of methanol to remove lipids. The quantity of 1,25(OH)_2_D_3_ was determined using the 1,25-dihydroxyvitamin D ^125^I RIA kit (INCSTAR, Stillwater, MN, USA). Briefly, 2 mL of water and 5 mL of methanol/water (70 : 30) were added to a C_18_OH cartridge to remove salts, polar lipids, and pigments under vacuum. Then, 5 mL of hexane/methylene chloride (90 : 10) was added to the C_18_OH cartridge to remove 25(OH)D_3_, and 5 mL of hexane/isopropanol (99 : 1) was added to remove 24,25(OH)_2_D_3_/25,25(OH)_2_D_3_. Each C_18_OH cartridge was tightly fitted inside a silica cartridge. After adding hexane/isopropanol (92 : 8) under vacuum, the C_18_OH cartridge was removed. Finally, purified 1,25(OH)_2_D_3_ was eluted from the silica cartridge in 5 mL of hexane/isopropanol (80 : 20). The levels of 1,25(OH)_2_D_3_ were quantitated by competitive radioimmunoassay (RIA) using ^125^I-labeled anti-1,25(OH)_2_D_3_ and anti-1,25(OH)_2_D_3_ antibodies.

### 2.6. Treatment with MTB Antigens

Monocytes (3 × 10^5^ cells/mL) were incubated with 10 *μ*g/mL MTB and 200 ng/mL 25(OH)D_3_. After 3 hours of incubation, 1 mL of acetonitrile was added to stop the reaction. The resulting 1,25(OH)_2_D_3_ was purified from the cells and the medium and quantitated by RIA. The remainder of the procedure was the same as for the previous assay.

### 2.7. Statistical Analysis

All data were analyzed using Student's *t*-test.

## 3. Results

### 3.1. Ionized Calcium and 1,25 Dihydroxyvitamin D_3_ Concentrations in a Patient with Active TB and Uremia

Serum ionized calcium and 1,25(OH)_2_D_3_ were determined at different time points from the first visit to one year after the completion of antituberculosis treatment. As shown in Figure 1, the levels of ionized calcium correlated with the concentration of 1,25(OH)_2_D_3_ (Figure 1).

### 3.2. Study Population

Twenty-five individuals were recruited into the frequent TB contact group and 25 patients into the active TB group. The mean age and sex ratio did not differ significantly between the groups. The characteristics of the dataset are presented in Table 1.

### 3.3. 1,25(OH)_2_D_3_ Quantitation

**Figure 2:**
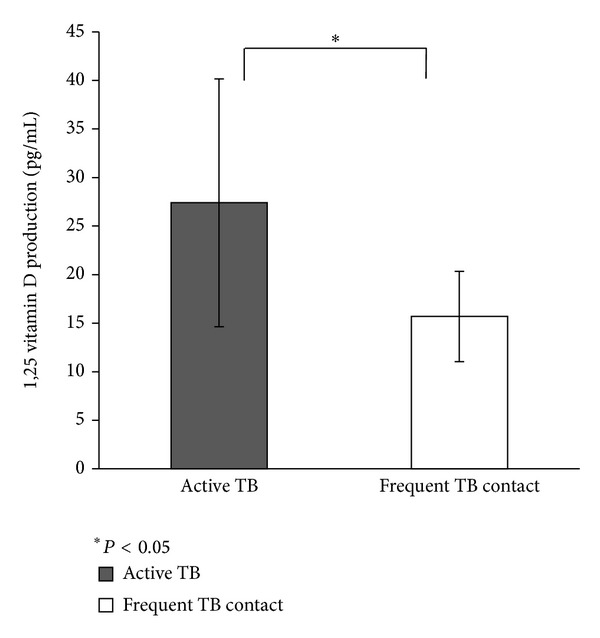
Quantitation of 1,25(OH)_2_D_3_ in patients with active TB and frequent TB contacts. RIA measurement of 1,25(OH)_2_D_3_ purified from monocyte suspensions cultured with 25(OH)D_3_ for 3 hours. The amount of 1,25(OH)_2_D_3_ in the active TB group was significantly higher than that in the frequent TB contact group. Each column represents the mean of 1,25(OH)_2_D_3_ quantitation. Error bars represent the standard deviation. *P < 0.05.

**Figure 3:**
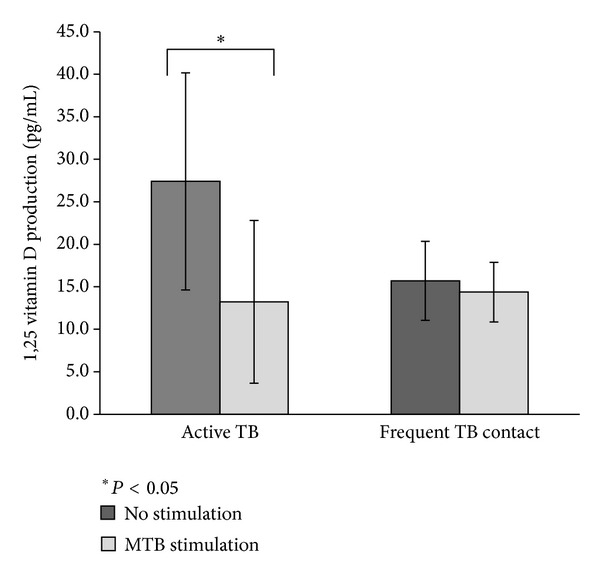
Quantitation of 1,25(OH)_2_D_3_ in patients with active TB and frequent TB contacts with or without *M. tuberculosis* (MTB) antigen. Monocytes were pulsed with 25(OH)D_3_ with or without MTB. The amount of 1,25(OH)_2_D_3_ with MTB exposure decreased significantly compared to that with no MTB in the active TB group. Each column represents the mean of 1,25(OH)_2_D_3_ quantitation. Error bars represent the standard deviation. *P < 0.05.

Monocytes were cultured with 25(OH)D_3_ for 3 hours. Then, 1,25(OH)_2_D_3_ was purified from the cells and the medium and measured by RIA. As shown in Figure 2, the amount of 1,25(OH)_2_D_3_ in the active TB group was 27.4 ± 12.8 pg/mL (mean ± SD), which was significantly higher than that in the frequent TB contact group (15.7 ± 4.7) (*P* < 0.05). When monocytes were simultaneously incubated with MTB and 25(OH)D_3_ for 3 hours, the amount of 1,25(OH)_2_D_3_ in the active TB group decreased significantly compared to that with no MTB (13.2 ± 9.6 and 27.4 ± 12.8, resp.) (*P* < 0.05) (see Figure 3). There was no difference between monocytes with or without exposure to MTB in the frequent TB contact group (14.4 ± 3.5 and 15.7 ± 4.7, resp.).

## 4. Discussion

We observed that 1,25(OH)_2_D_3_ levels correlated with ionized calcium levels in a uremic patient with pulmonary tuberculosis. In active pulmonary tuberculosis, the serum levels of 1,25(OH)_2_D_3_ in this patient were high, which in turn induced high levels of ionized calcium. After treatment, both 1,25(OH)_2_D_3_ and ionized calcium levels decreased. Theoretically, in a uremic patient, renal CYP27B1 activity is trivial. Low serum 1,25(OH)_2_D_3_ levels are often observed in uremic patients [12]. Consistent with what has been observed in humans, in an anephric mouse model, low 1,25(OH)_2_D_3_ levels are also observed [13]. However, an extrarenal source of CYP27B1 has been described for more than 6 decades. Harrell and Fisher were among the first to find extrarenal synthesis of 1,25(OH)_2_D_3_ under pathological conditions [14]. These authors established the association between dysregulated calcium homeostasis and sarcoidosis. Tissue macrophages were later shown to be an extrarenal source of 1,25(OH)_2_D_3_ production in these patient groups [15]. Increasing numbers of tissues were found to express CYP27B1 by different study groups, such as skin melanocytes, tissue macrophages, and residual cells of the placenta [11]. Surprisingly, not every cell that expresses CYP27B1 possesses enzymatic activity [16]. To clarify the potential for circulating monocytes to contribute to high 1,25(OH)_2_D_3_ levels, we utilized an ex vivo bioassay using 25(OH)D_3_ as a substrate to determine the activity of CYP27B1. Our results demonstrate that CYP27B1 activity in monocytes from patients with active TB is significantly higher than that in monocytes from individuals with frequent TB contact. Circulating monocytes contribute to the conversion of 25(OH)D_3_ to the more active 1,25(OH)_2_D_3_. Intriguingly, vitamin D_3_ was traditionally considered to be an endocrine factor; 1,25(OH)_2_D_3_ produced at local sites (renal tubules) is carried by the blood to affect other tissues or organs, for example, bone. However, in recent decades, vitamin D_3_ was shown to play roles other than endocrine functions. The 1,25(OH)_2_D_3_ produced by inflammatory cells can stimulate vitamin D receptor expression in both neighboring cells and in the inflammatory cells themselves. By binding to the vitamin D receptor and the retinoid receptor, the ligand-receptor complex can bind to the promoters of many inflammatory genes [17, 18]. Because of these effects, vitamin D_3_ was considered to have both paracrine and autocrine functions. In contrast with the endocrine function of vitamin D_3_ as a calcium regulator, its paracrine and autocrine functions induce inflammatory cells to produce antibacterial peptides and augment the process of autophagy [19–23]. Because 1,25(OH)_2_D_3_ is produced locally and is not carried to target sites for the regulation of calcium homeostasis, its production does not affect serum calcium levels. Interestingly, in this study, we found that 1,25(OH)_2_D_3_ produced by monocytes was a special case. Although 1,25(OH)_2_D_3_ produced by monocytes can act locally, these cells are carried by the blood to tissues throughout the body. The 1,25(OH)_2_D_3_ produced by monocytes also behaves as an endocrine factor. Monocytes may be the only cells in our bodies that can orchestrate the endocrine, paracrine, and autocrine functions of vitamin D_3_. An interesting issue is what is the relative contribution of monocyte source of 1,25(OH)_2_D_3_ to total 1,25(OH)_2_D_3_ level. Dusso et al. observed that maximal production of 1,25(OH)_2_D_3_ from monocyte is trivial (in fmole/hour/microgram DNA) compared to 1,25(OH)_2_D_3_ concentration in normal (in pmol/mL) [24]. But the data is from an ex vivo experiment, we do not know how many monocytes in the body are stimulated to produce 1,25(OH)_2_D_3_. We speculate that the relative contribution of monocyte source of 1,25(OH)_2_D_3_ to total 1,25(OH)_2_D_3_ level is small in most circumstances. Even in patients with granulomatous disease, only few of them have clinical symptoms of hypercalcemia resulting from high level of 1,25(OH)_2_D_3_. Our indicator case is one of them.

 We also found that when monocytes from patients with active TB are cultured with MTB antigen, 1,25(OH)_2_D_3_ conversion is significantly lower than when no antigen is added. In the frequent TB contact group, there was no difference between being with or without MTB antigen. There are two possible explanations for this observation. First, primed monocytes from patients with active TB can induce more 24(OH) hydroxylase (CPY24) activity than their counterparts, which actively hydroxylates 1,25(OH)_2_D_3_ to calcitroic acid, when they are further stimulated with MTB antigen. vitamin D_3_ induces not only inflammatory gene products but also the expression of CPY24 [25, 26]. CPY24 is a major catabolic enzyme of vitamin D_3_. CYP27B1 and CYP24 activities are modulated in a diametrically opposite way to control serum levels of 1,25(OH)_2_D_3_ [26]. Owing to this concerted action, hypercalcemia is seldom observed in patients. The frequent TB contact group demonstrated an even better concerted action, which is why there was no change in 1,25(OH)_2_D_3_ conversion after stimulation with MTB antigen. A second explanation is that CPY27B1 activity is exhausted when monocytes are restimulated by MTB antigen.

mRNA expression was not determined in this study for the following reasons. First, a large number of monocytes from participants were required for the ex vivo bioassay. If we performed both quantitative mRNA expression and the ex vivo bioassay, we would have needed to collect more than 30 mL of blood from each participant. The collection of this volume of blood was rejected by our ethical committee. Second, as indicated previously, some tissues may express CYP27B1 without detectable enzymatic activity. mRNA levels are not always correlated with enzymatic activity. Despite CYP27B1 expression in some tissues, no enzymatic activity was detected. In future studies, the levels of CYP27B1 and CYP24 and the metabolites of vitamin D_3_, such as calcitroic acid and 24,25(OH)_2_D_3_, should be quantified to clarify vitamin D_3_ metabolism in the pathophysiological process of tuberculosis. 

## 5. Conclusion

In conclusion, calcium levels correlated with 1,25(OH)_2_D_3_ levels in a uremic patient infected with tuberculosis. Furthermore, we found that CYP27B1 activity in monocytes is higher among patients with active tuberculosis than those with frequent TB contact.

## Figures and Tables

**Table 1:** Sex and age of study participants.

	Active TB (*N* = 25)	Frequent TB contact (*N* = 25)	*P* value
Sex			0.47
Male	13 (52%)	12 (48%)	
Female	12 (48%)	13 (52%)	
Age (mean ± SD)	44 ± 6.8	40 ± 6.2	
^ a^Duration of disease (months, mean ± SD)	3 ± 1.2		
^ b^Duration of exposure (years, mean ± SD)		13 ± 2.4	

^a^Duration of disease was defined as the interval from diagnosis to blood collection.

^b^Duration of exposure was defined as the interval from beginning work in a TB center to blood collection.
